# In vitro biological responses to nanofibrillated cellulose by human dermal, lung and immune cells: surface chemistry aspect

**DOI:** 10.1186/s12989-016-0182-0

**Published:** 2017-01-10

**Authors:** Viviana R. Lopes, Carla Sanchez-Martinez, Maria Strømme, Natalia Ferraz

**Affiliations:** 1 Nanotechnology and Functional Materials, Department of Engineering Sciences, Uppsala University, Box 534, 75121 Uppsala, Sweden; 2 Present affiliation: Ocular Biology and Therapeutics, UCL Institute of Ophthalmology, 11-43 Bath Street, EC1V 9EL London, UK

**Keywords:** Cytotoxicity, Inflammation, Nanocellulose, Surface chemistry

## Abstract

**Background:**

Nanocellulose, and particularly nanofibrillated cellulose (NFC), has been proposed for a diversity of applications in industry and in the biomedical field. Its unique physicochemical and structural features distinguish nanocellulose from traditional materials and enable its use as an advance nanomaterial. However, its nanoscale features may induce unknown biological responses. Limited studies with NFC are available and the biological impacts of its use have not been thoroughly explored. This study assesses the in vitro biological responses elicited by wood-derived NFC gels, when human dermal fibroblasts, lung MRC-5 cells and THP-1 macrophage cells are exposed to the nanomaterial. Furthermore, whether the presence of surface charged groups (i.e. carboxymethyl and hydroxypropyltrimethylammonium groups) on NFC can induce distinct biological responses is investigated.

**Results:**

The introduction of surface charged groups resulted in individual nanofibrils, while fibril aggregates predominated in the unmodified NFC gel suspensions as observed by transmission electron microscopy. In the presence of proteins, the surface modified NFCs formed compact agglomerates while the agglomeration pattern of the unmodified NFC was similar in the presence of proteins and in physiological buffer. Unmodified and modified NFC gels did not induce cytotoxicity in human dermal fibroblasts, lung and macrophage cells. No significant ROS production by THP-1 macrophages was found and no cellular uptake was observed. However, an inflammatory response was detected when THP-1 macrophages were treated with unmodified NFC as assessed by an increase in TNF-α and IL1-β levels, an effect that was absent when surface charged groups were introduced into NFC.

**Conclusions:**

Taken together, the data presented here show the absence of cytotoxic effects associated with the exposure to unmodified, carboxymethylated and hydroxypropyltrimethylammonium-modified NFCs. Unmodified NFC presented a pro-inflammatory effect which can be further moderated by introducing surface modifications such as carboxymethyl and hydroxypropyltrimethylammonium groups into the nanofibrils. The present findings suggest that the inflammatory response to NFC might be driven by the material surface chemistry, and thus open up for the possibility of designing safe nanocellulose materials.

**Electronic supplementary material:**

The online version of this article (doi:10.1186/s12989-016-0182-0) contains supplementary material, which is available to authorized users.

## Background

In recent years, an emerging demand for nano-based products from sustainable and environmental-friendly resources is placing nanocellulose on top of exciting nanomaterials near commercialization [1–4]. Nanocellulose consists of cellulose fibrils or crystallites with at least one dimension in the nanoscale and presents the typical physicochemical properties of cellulose such as hydrophilicity, mechanical strength and broad possibility of chemical modifications together with specific nanomaterial properties like high specific surface area and high aspect ratio [3, 4]. It can be derived from a diversity of sources, including wood, algae, bacteria and tunicates. Nanocellulose from wood and other higher plants is typically isolated as crystals (nanocellulose crystals) or nanofibrils (nanofibrillated cellulose (NFC)) through chemical and/or mechanical treatment of cellulose, while fibres of bacterial nanocellulose are produced by bacterial synthesis from low molecular weight sugars or other carbon sources [3–5].

Due to its novel physicochemical and structural features, nanocellulose has been proposed for a myriad of applications in industry (paper, packaging, electronics, cosmetics, etc.) and in the biomedical field, such as sensors and scaffolds for tissue engineering [6–10]. Although cellulose is considered as non-toxic, the novel physicochemical properties and nanoscale dimensions of nanocellulose may imply different biological effects from conventional cellulose [4, 5].

With the expected increase in the presence of nanocellulose in consumer products it is critical to assess and confirm the safety of the nanomaterial [8, 11]. Particularly, from an occupational point of view, potential hazard effects have been recently identified during the different life cycle stages of nanocellulose [8]. During production and manufacturing of nanocellulose, two main potential hazards were identified, i) accidental inhalation of nanocellulose released to air after drying it and packaging it, and ii) dermal contact with slurry of nanocellulose when it is being combined with other materials, which can spill to the skin or clothes of the worker [8].

There is a void of knowledge concerning the effect of cellulose-based nanomaterials on human health [8]. The in vitro studies available for nanocellulose, particularly for NFC, are mostly focus on exposure by inhalation with data from exposure via the dermal and oral routes still lacking [8]. The few toxicological studies with NFC have so far shown no indication of toxicity [12–15]. However, different raw materials, manufacturing process and post-manufacturing chemical modifications may alter the material’s physicochemical properties [16]. Properties like fibril dimensions, degree of crystallinity, specific surface area, degree of branching of the nanofibrils and modifications of the material chemical properties may affect the interactions between the cellulose nanofibrils and biological systems.

In the work presented here the focus is on NFC derived from wood and the potential occupational hazards derived from exposure to it. The aim is to evaluate the in vitro biological responses elicited by NFC, having in mind an occupational scenario and focusing on inhalation and dermal exposure routes. Whether chemical surface modifications of NFC could cause distinct biological responses is further investigated. Typically, surface charged groups are introduced during NFC production to facilitate the fibrillation process by adding repulsive charges [11]. In this context, a side-by-side in vitro toxicity assessment of wood-derived NFC gels with different surface modifications (carboxymethylated and hydroxypropyltrimethylammonium-modified NFC) towards human dermal fibroblasts (HDF), lung MRC-5 cells and THP-1 macrophage cells is presented here, together with the evaluation of the cell responses to unmodified NFC.

## Methods

### Synthesis and surface modification of NFC

NFC was produced from commercial, never dried, bleached sulfite softwood dissolving pulp (Domsjö Fabriker AB, Sweden). Unmodified-NFC (U-NFC), carboxymethylated-NFC and hydroxypropyltrimethylammonium-NFC, here referred to as anionic NFC (A-NFC) and cationic NFC (C-NFC), in that order, were provided by Innventia AB (Sweden). U-NFC was prepared by enzymatic pretreatment of the pulp [17] while carboxymethylation and epoxypropyltrimethylammonium chloride (EPTMAC) quaternization pretreatments were used to prepare A-NFC and C-NFC respectively, as previously described [18]. All samples were biocide free and showed no bacterial contamination when tested with 3 M™ Petrifilm™ bacteria tests (total aerobic count).

### NFC characterization

#### Transmission electron microscopy

The morphology of the fibres in phosphate buffer and in cell culture medium was investigated by transmission electron microscopy (TEM). Samples were prepared as described by Usov et al. [19]. Briefly, 5 μl of NFC stock solution (in PBS) were deposited onto carbon-coated copper grids (400 mesh), while 5 μl of NFC in RPMI cell culture medium (500 μg/ml, prepared as described below) were placed on formvar coated 200 mesh copper grids. After adsorption, the sample grids were stained with 2% uranyl acetate to achieve a noncrystalline film of stain embedding the fibres. Following the staining step, the excess moisture was drained along the periphery using filter paper. Dried grids were examined using TEM (FEI Tecnai G2) operated at 100 kV or at 80 kV.

### ζ – potential

Dispersions of 0.001% (w/w) of the NFC samples were prepared in 10 mM NaCl and in cell culture media, DMEM and RPMI1640 (ThermoFisher Scientific), through ultrasonication for 30 s (Vibracell 600 W, 20 kHz). The electrophoretic mobility of the samples was measured using a universal dip cell in a ZetaSizer Nano instrument (Malvern Instruments) at 25 °C and 37 °C (in the case of cell culture media). The ζ-potentials were determined from the electrophoretic mobility applying the Smoluchowski equation [20].

### Preparation of NFC exposure suspensions

Stock solutions of U-NFC, A-NFC and C-NFC were prepared in phosphate buffer (PBS) at 5 mg/mL and dispersed with an ultrasonic probe (Vibracell 600 W, 20 kHz) during 12 min. The final stock solutions were sterilized by autoclaving, except for C-NFC which was subject to ultraviolet radiation (UV) treatment during two cycles of 45 min each. Before each exposure, the stock solutions were diluted in cell culture media (concentration range 50 to 500 μg/mL) and sonicated for 30 min in a water bath sonicator (Bransonic 3510) before being added to the cells.

### Cell culture

The human dermal fibroblasts (HDF) from European Collection of Authenticated Cell Cultures (ECACC), the human MRC-5 lung fibroblast cell line (private collection), and the human THP-1 monocytic cell line (ECACC) were used in this study. MRC-5 and HDF cells were cultured in DMEM/F12 medium (ThermoFisher Scientific), and THP-1 cells in RPMI 1640 medium (ThermoFisher Scientific) supplemented with 10% (v/v) heat-inactivated fetal bovine serum (FBS), 100 IU/mL penicillin and 100 μg/mL streptomycin (both from ThermoFisher Scientific). The cells were incubated in a humidified atmosphere at 37 °C, 5% CO_2_ and sub-cultured at 70–80% confluency.

### Cytotoxicity assessment

For each experiment, HDF and MRC-5 cells were seeded in 96 well-plates at a density of 6 × 10^3^ cells/well (200 μl/well) 1 day prior to NFC treatment. THP-1 monocytes were seeded in 96 well-plates at a density of 2.5 × 10^4^ cells/well (200 μl/well) and differentiated into macrophages using 5 ng/mL phorbol myristate acetate (PMA) (Sigma-Aldrich) for 48 h before NFC treatment [21].

Near confluent monolayers of HDF and MRC-5 cells, and THP-1 differentiated macrophages were finally exposed to U-NFC, A-NFC and C-NFC at concentrations ranging from 50 to 500 μg/mL in 96-well plate (200 μl/well) for 24 h. Food grade microcrystalline cellulose (MCC, Avicel® CL-611, 100 nm in diameter microcrystals mixed with soluble sodium carboxymethylcellulose, FMC Biopolymer) was used as reference material. As a positive control, 5% (w/w) dimethyl sulfoxide (DMSO) (Sigma-Aldrich) in cell culture medium was used, while non-treated cells served as negative control.

### Cellular metabolic activity: Alamar Blue assay

The alamar blue (AB) assay was used to assess cellular metabolic activity as a marker of cytotoxicity. After exposure to NFC, cells were carefully washed with 100 μL warm PBS, and 200 μL of Alamar Blue® reagent (ThermoFisher Scientific) diluted (1:10) was added to each well and incubated for 90 min at 37 °C. After incubation, 100 μL of each well were collected and added to a black 96-well plate. The fluorescence was measured at 560 nm excitation and 590 nm emission wavelengths using a plate reader (Tecan Infinite M200). Results were expressed as percentage of cell viability with respect to the negative control. The experiments were performed at least three times in triplicate wells for each dose. Interference of the NFC samples with the assay was tested in an acellular system by incubating different doses of NFC with the AB reagent for 90 min at 37 °C in 96 well plates. Cell culture media interference was also measured. Neither NFC samples nor the cell culture media interfere with the AB assay.

### Cellular membrane integrity: Lactate Dehydrogenase assay

The release of the intracellular enzyme lactate dehydrogenase (LDH), indicative of cell membrane damage, was assessed by a LDH kit (Abcam) according to the manufacturer’s guidelines. Briefly, after cell exposure to the NFC samples, cell culture supernatants were collected and adherent cells were lysated for 30 min with cell lysis buffer diluted in cell culture medium (1:10) and the lysates were collected. Lysates and supernatants were centrifuged at 6800 *g* during 10 min to avoid any potential interference of NFC. The enzyme activity in the lysates and supernatants samples was measured by reading the absorbance at 450 nm wavelength and reference wavelength 650 nm using a plate reader (Tecan Infinite M200). The experiments were conducted at least three times in duplicate wells for each dose. LDH release (LDH activity in cell culture supernatant) was normalized by the total LDH activity (sum of LDH activity in cell culture supernatants and lysates) which correlates with the total number of cells in order to avoid any underestimation of toxicity [22].

### Cell morphology - Light microscopy

After 24 h exposure to NFC, cells were carefully rinsed with warm PBS and observed under light microscopy (Nikon Eclipse TE2000-U) to evaluate their morphology.

### Inflammation assessment

The inflammatory response was investigated by quantifying the secreted levels of the cytokines tumor necrosis factor α (TNF-α) and interleukin 1 beta (IL1-β). THP-1 monocytes were differentiated into macrophages and treated with the NFC suspensions as described above. After 24 h exposure, cell culture supernatants were collected, centrifuged at 6800 *g* during 10 min and further analyzed for the levels of cytokines using ELISA Kits (human TNF-α and human IL1-β ELISA Kits, Thermo Fischer Scientific) according to the manufacturer’s protocol. As a positive control for TNF-α and IL1-β induction, cells were treated with lipopolysaccharide (LPS) from *Pseudomonas aeruginosa* at 1 ng/mL. The same experiments were performed in the presence of polymyxin B (PMB) at a final concentration of 25 μg/mL in order to inhibit the potential effects of any endotoxin present in the NFC samples [23]. The experiments were conducted at least three times in duplicate wells for each dose. TNF-α and IL1-β concentrations were calculated from a standard curve plotted for each experiment.

### Reactive oxygen species production

The levels of intracellular reactive oxygen species (ROS) were measured using the dichlorodihydrofluorescein diacetate (DCFH-DA) assay (Abcam) according to the manufacturer’s guidelines. DCFH-DA is a lipophilic cell permeable compound that is deacetylated in the cytoplasm by cellular esterases, and later oxidized by ROS to a highly fluorescent molecule. THP-1 monocytes were differentiated into macrophages and loaded with 20 μM DCFH-DA in PBS for 30 min at 37 °C. Thereafter, cells were treated with the NFC suspensions (50, 100, 250 500 μg/mL) and fluorescence was recorded every 30 min over 120 min (excitation 485 nm, emission 535 nm) at 37 °C using a plate reader (Tecan Infinite M200). Tert-butyl hydroperoxide (TBHP, 50 μM) was used as positive control.

### Cellular uptake of NFC - Transmission electron microscopy

TEM was used to investigate if the NFC materials were uptaken by THP-1 macrophages. THP-1 macrophages were exposed to the different NFC samples (500 μg/ml) for 24 h and then fixed in 2.5% (v/v) glutaraldehyde overnight at 4 °C. Samples were washed with sodium cacodylate buffer and subsequently post-fixed with 1% osmium tetroxide in sodium cacodylate buffer. Afterwards, the cells were dehydrated in ascending ethanol series, embedded in epon and finally polymerized at 60 °C for 48 h. From the embedded cells, ultrathin sections (50–60 nm) were cut parallel to the vertical axis of the inserts, mounted on copper grids and stained with uranyl acetate and lead citrate. Imaging was done with a Technai G2 microscope (FEI, Netherlands) LaB6 filament at 80 kV.

### Statistical analysis

Data analysis was conducted using GraphPad Prism 6, version 6.07 (GraphPad Software Inc., La Jolla, USA) by one-way or two-way analysis of variance (ANOVA) followed by Dunnett’s multiple comparison post-*hoc* tests. *p*-values lower than 0.05 were considered statistically significant. Results are presented as the mean ± standard error of the mean (SEM).

## Results

### NFC characterization

A detailed physico-chemical characterization of the NFC samples evaluated in this study is summarized in Table 1.

**Table 1:** Physico-chemical characteristics of the NFC samples under study

					ζ-potential (mV)		
NFC	Surface modification	Content of functional groups (mmol/g) ^a^	Degree of crystallinity^b^ (%)	Fibre diameter (in PBS) ^c^	NaCl ^d^	DMEM ^e^	RPMI1640 ^f^
U-NFC	none	0.03^g^	36	20–30 nm fibril aggregates	−11.3 ± 1.3	−4.0 ± 0.2	−5.2 ± 0.6
A-NFC	Carboxy-methylation	0.53 ^g^	32	4–5 nm individual fibrils	−34.1 ± 1.7	−12.7 ± 2.7	−12.6 ± 1.6
C-NFC	EPTMAC quaternization	1.60 ^h^	32	4–5 nm individual fibrils	24.2 ± 2.0	−7.2 ± 2.9	−8.1 ± 2.0

^a^ Data from [24]

^b^ Determined by XRD measurements from previous study [18]

^c^ Determined by TEM

^d^ Determined in 10 mM NaCl at 25 °C and at pH 7.4–7.5

^e^ Determined at 37 °C and at pH 7.5–7.8

^f^ Determined at 37 °C and at pH 7.9–8.1

^g^ Carboxyl groups

^h^ Hydroxypropyltrimethylammonium groups

The successful introduction of carboxymethyl and hydroxypropyltrimethylammonium groups on A-NFC and C-NFC, respectively, was previously verified by Hua et al. [24]. Results showed a higher surface group density for C-NFC than for A-NFC. No specific surface groups were introduced during the production of U-NFC (mild enzymatic pretreatment of the wood pulp) and thus only low levels of carboxyl group content could be expected due to the presence of residual hemicellulose. Furthermore, the degree of crystallinity, as previously determined [18], was similar between both unmodified and modified NFCs indicating that the chemical modifications did not considerably alter the crystallinity of NFC.

Zeta potential was measured for all NFCs. At pH 7.4, A-NFC and C-NFC suspended in 10 mM NaCl presented zeta potential values of − 34.1 and 24.2 mV, respectively, confirming the presence of the negatively and positively charged groups in the modified NFCs. The high absolute values of zeta potential for the charged samples implies a good stability of the suspensions [25]. However, U-NFC formed an unstable dispersion, showing tendency to aggregate, with a slightly negative surface charge (−11.3 mV). When incubated in cell culture media and at 37 °C, all NFCs samples showed negative zeta potential values in the range of − 4 and − 12 mV, independently of the cell culture medium used.

**Fig. 1:**
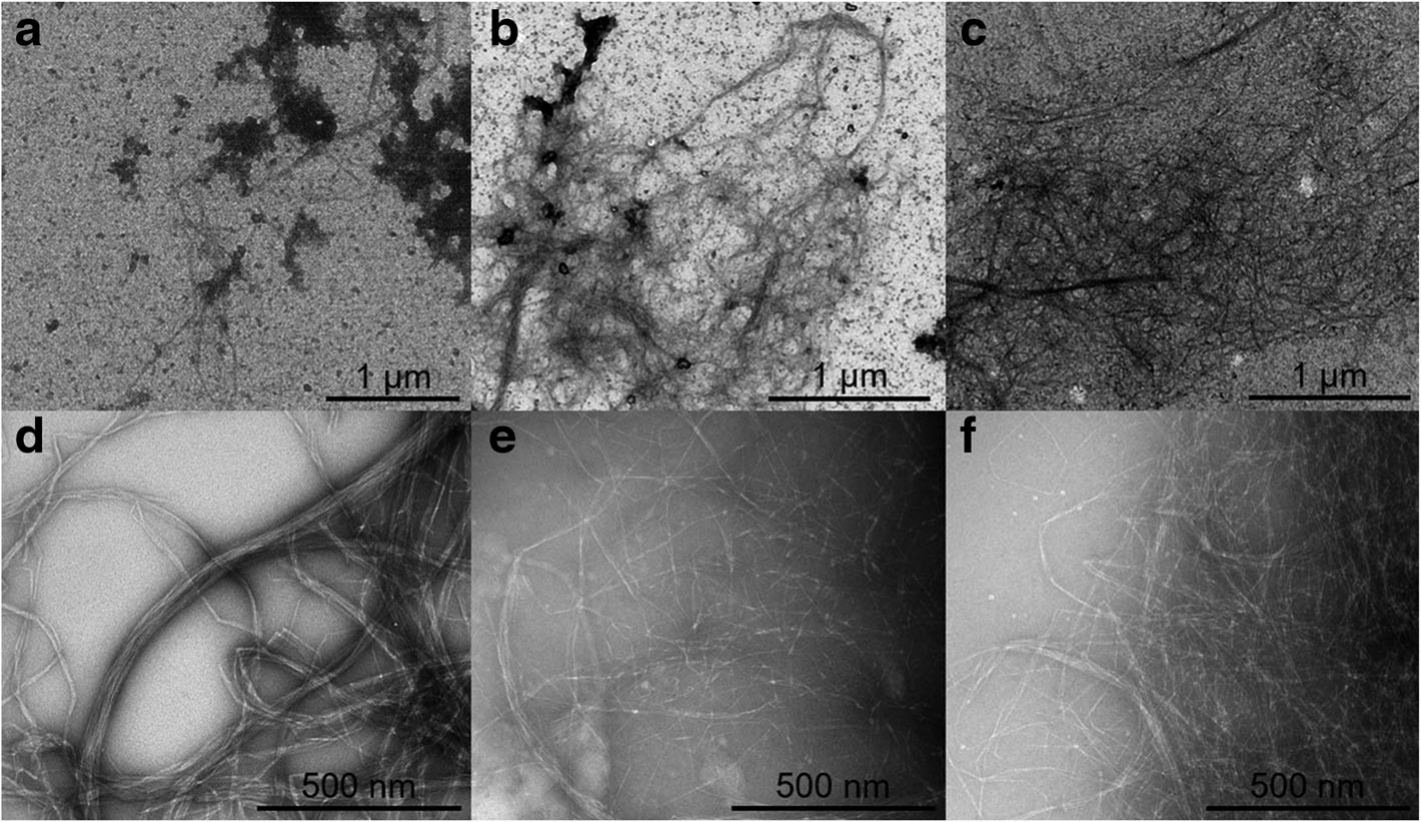
Morphology of NFC suspended in phosphate buffer. Transmission electron microscopy images of U-NFC (**a** and **d**), A-NFC (**b** and **e**) and C-NFC (**c** and **f**)

The morphology of the nanocellulose fibres suspended in PBS was observed by TEM (Fig. 1). Small agglomerates were observed in U-NFC, while A-NFC and C-NFC suspensions showed dispersed fibres (Fig. 1, upper panels). High magnification images (Fig. 1, lower panels) depict the dimensions of the individual fibrils or fibril aggregates. U-NFC showed bundles of several μm long fibres, forming 20–30 nm in diameter fibre aggregates (Fig. 1d). The presence of surface charges on A-NFC and C-NFC resulted in better dispersion of the individual fibres, as shown in Fig. 1e and f, where individual nanofibrils (4–5 nm in diameter) with slight aggregation can be observed.

**Fig. 2:**
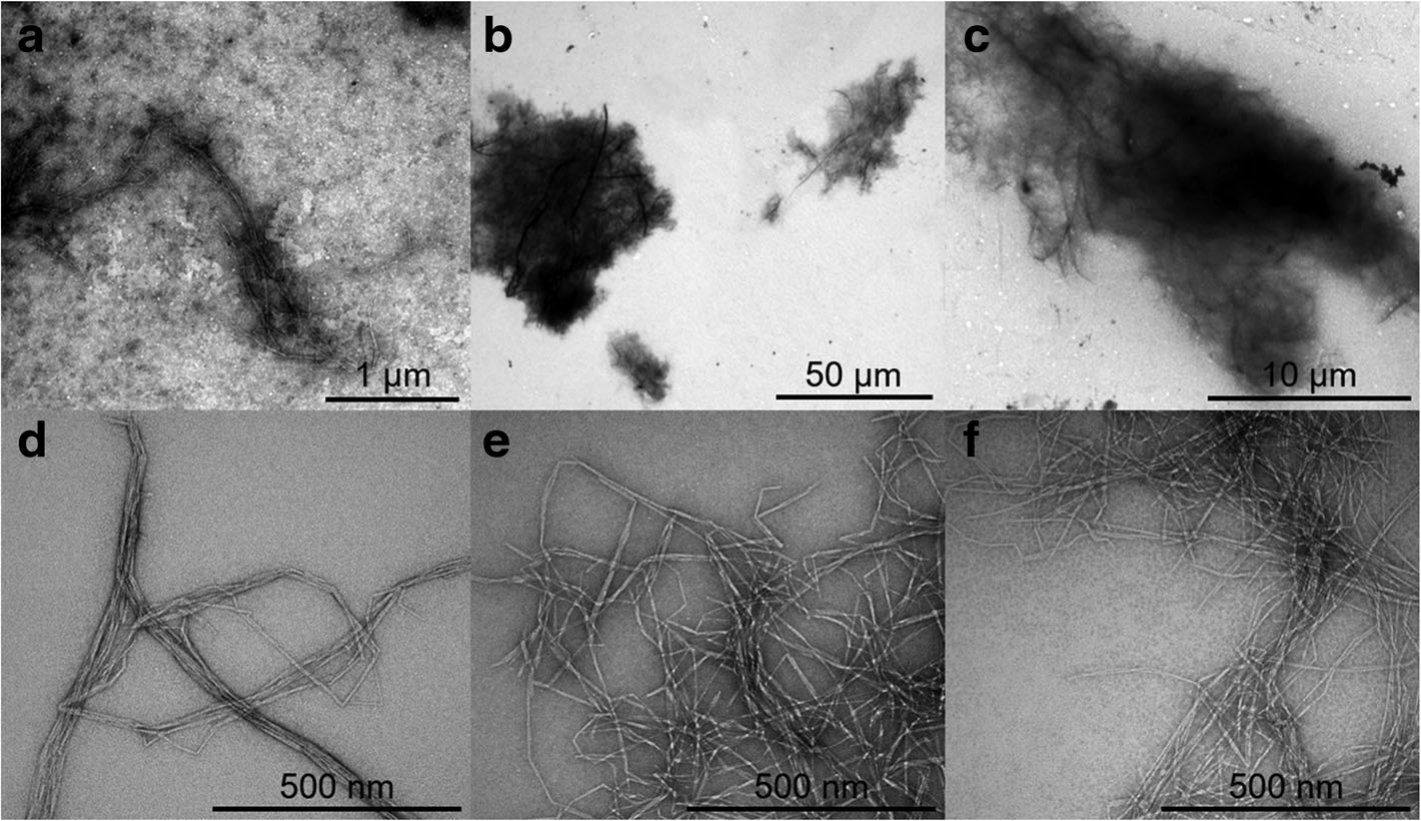
Morphology of NFC suspended in cell culture medium. Transmission electron microscopy images of U-NFC (**a** and **d**), A-NFC (**b** and **e**) and C-NFC (**c** and **f**)

When the NFC suspensions in cell culture medium were observed under TEM, compact agglomerates 10–50 μm in diameter were observed in the A-NFC and C-NFC samples, while smaller agglomerates (1–2 μm in diameter) were found in the U-NFC suspension (Fig. 2, upper panels). When taking a closer look at the agglomerates (Fig. 2, lower panels), it was observed that A-NFC and C-NFC still presented individual nanofibrils and the U-NFC sample had fibre aggregates similar to the ones observed when suspended in PBS.

### NFCs are not cytotoxic for immune, dermal and lung cells

**Fig. 3:**
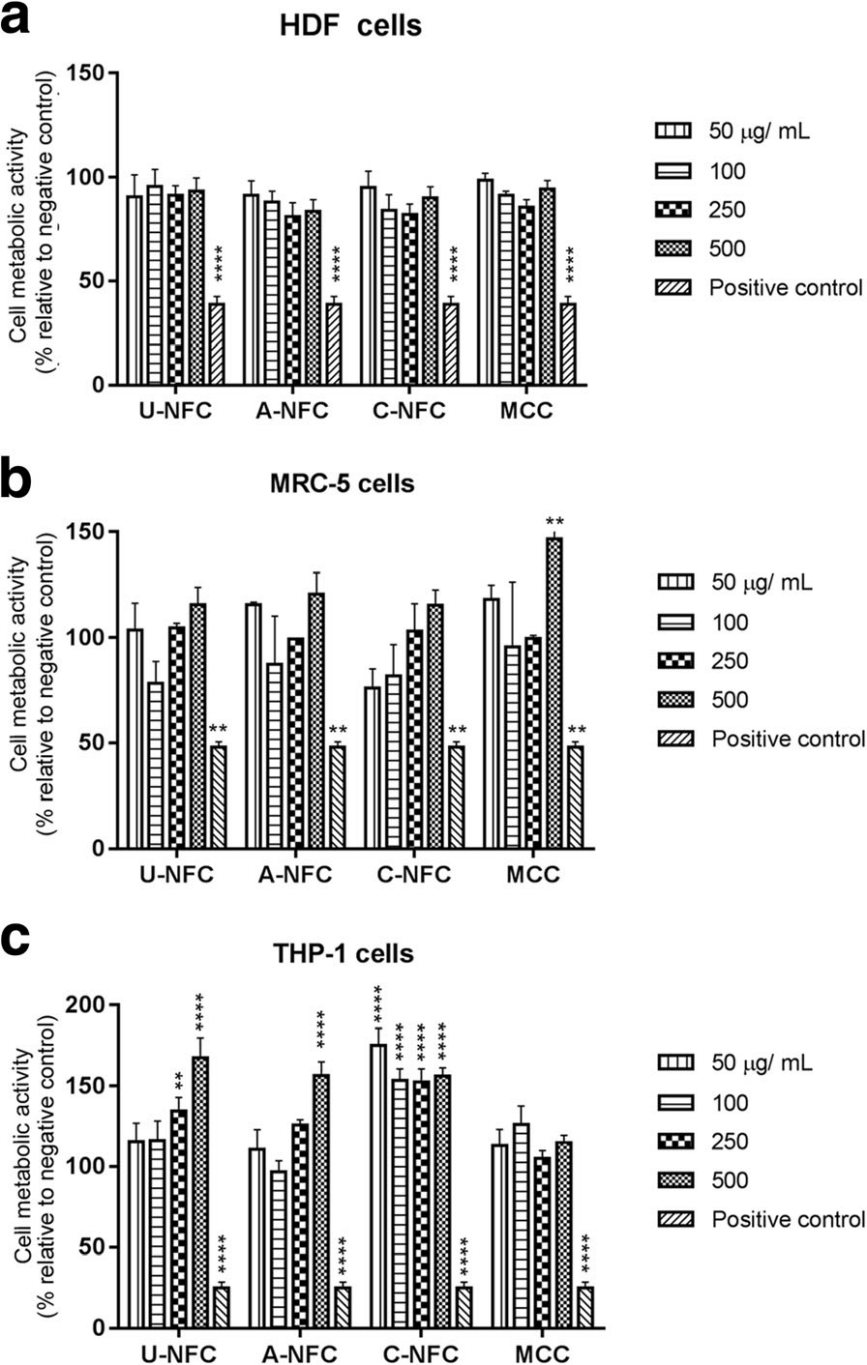
Metabolic activity of cells after NFC exposure. Cell viability of NFC-treated cells was assesed by evaluating cell metabolic activity using the alamar *blue* assay. **a** HDF cells, **b** MRC-5 cells and **c** THP-1 macrophages exposed to increasing doses of NFC (50–500 μg/mL) for 24 h. MCC is a food grade nanocellulose used as a reference material. The positive control was DMSO (5% v/v in cell culture media) an inducer of cytotoxic effects. Data are expressed as percentage relative to the negative control (untreated cells) and presented as mean ± SEM of three independent experiments. Significant results as compared to the negative control are marked with asterisks (** *p* < 0.01 and **** *p* < 0.0001)

**Fig. 4:**
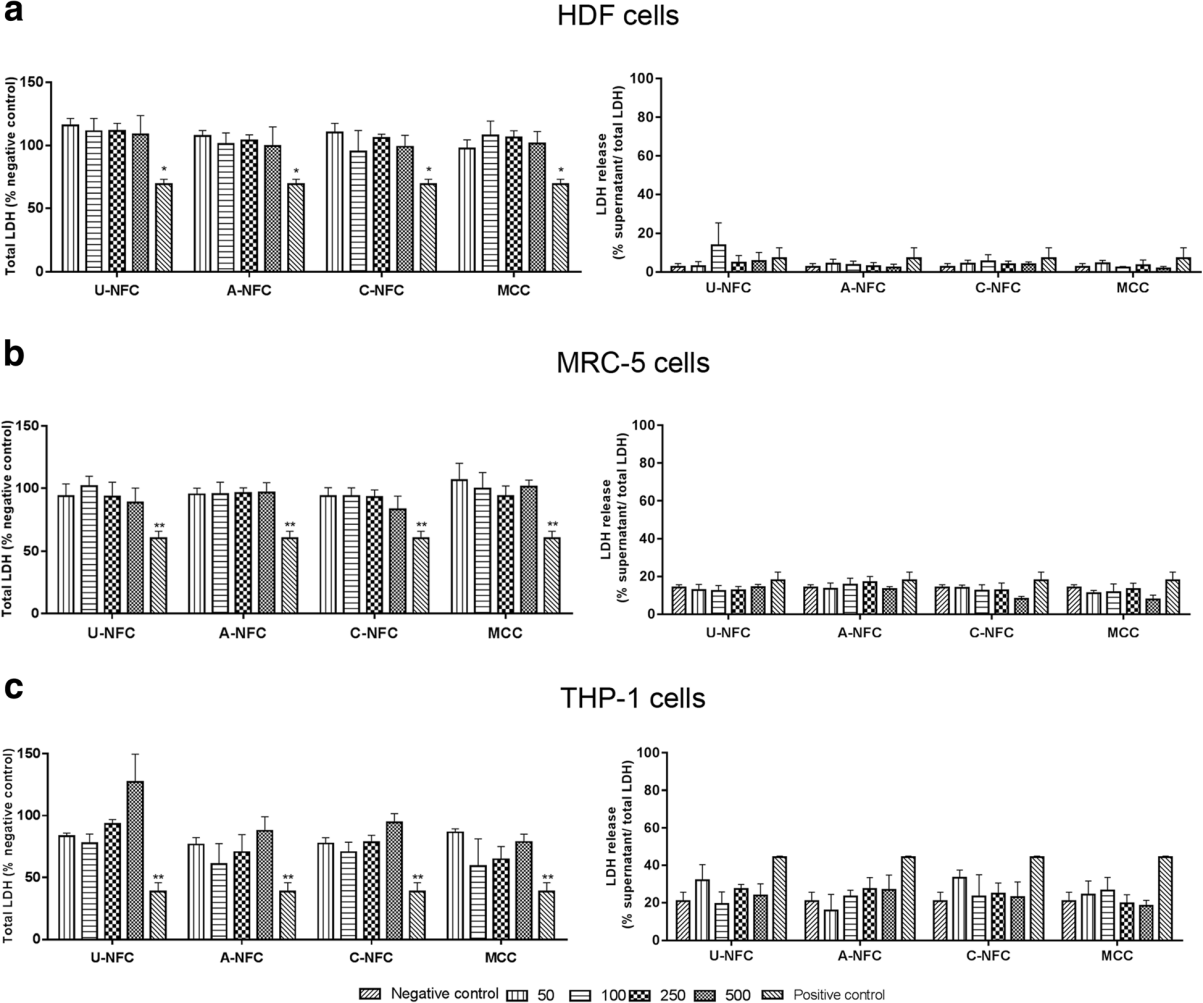
Lactate dehydrogenase (LDH) activity of cells after NFC exposure. Cytotoxicity of NFC-treated cells was evaluated by measuring total and extracellular LDH activity. **a** HDF cells, **b** MRC-5 cells and **c** THP-1 macrophages were treated with a range of NFC concentration from 50 to 500 μg/mL during 24 h. MCC is a food grade nanocellulose used as a reference material. The positive control was DMSO (5% v/v in cell culture media) an inducer of cytotoxicity. Data are presented as mean ± SEM of three independent experiments. Significant results as compared to the negative control are marked with asterisks (* *p* < 0.05 and ** *p* < 0.01)

The cytotoxic effect of the different NFCs was evaluated by using two different assays, AB and LDH assays, i.e. by investigating the effect on cell metabolic activity and on cell membrane integrity, respectively (Figs. 3 and 4).

The AB assay showed that after 24 h exposure, the metabolic activity of the treated cells was comparable to the activity of the non-treated cells (negative control) for the HDF and MRC-5 cells (Fig. 3a and b). Interestingly, THP-1 cells showed a significantly higher metabolic activity of the NFC-treated cells compared with the negative control (Fig. 3c), an effect that has been also observed by other authors when exposing macrophages to NFC [15].

No signs of toxicity were observed when the LDH assay was used to evaluate cell membrane damage following 24 h treatment with NFCs (Fig. 4). HDF cells exposed to NFCs with or without surface modifications did not significantly change the LDH release when compared to the negative control (Fig. 4a). Moreover, no significant differences in total cell number (total LDH) between treated and non-treated cells were found. The MRC-5 cells and THP-1 macrophages showed a similar pattern of LDH release and total LDH to the results found for HDF cells (Fig. 4b and c, respectively). The reference material MCC was also not cytotoxic for the studied cells under the conditions of the present work.

**Fig. 5:**
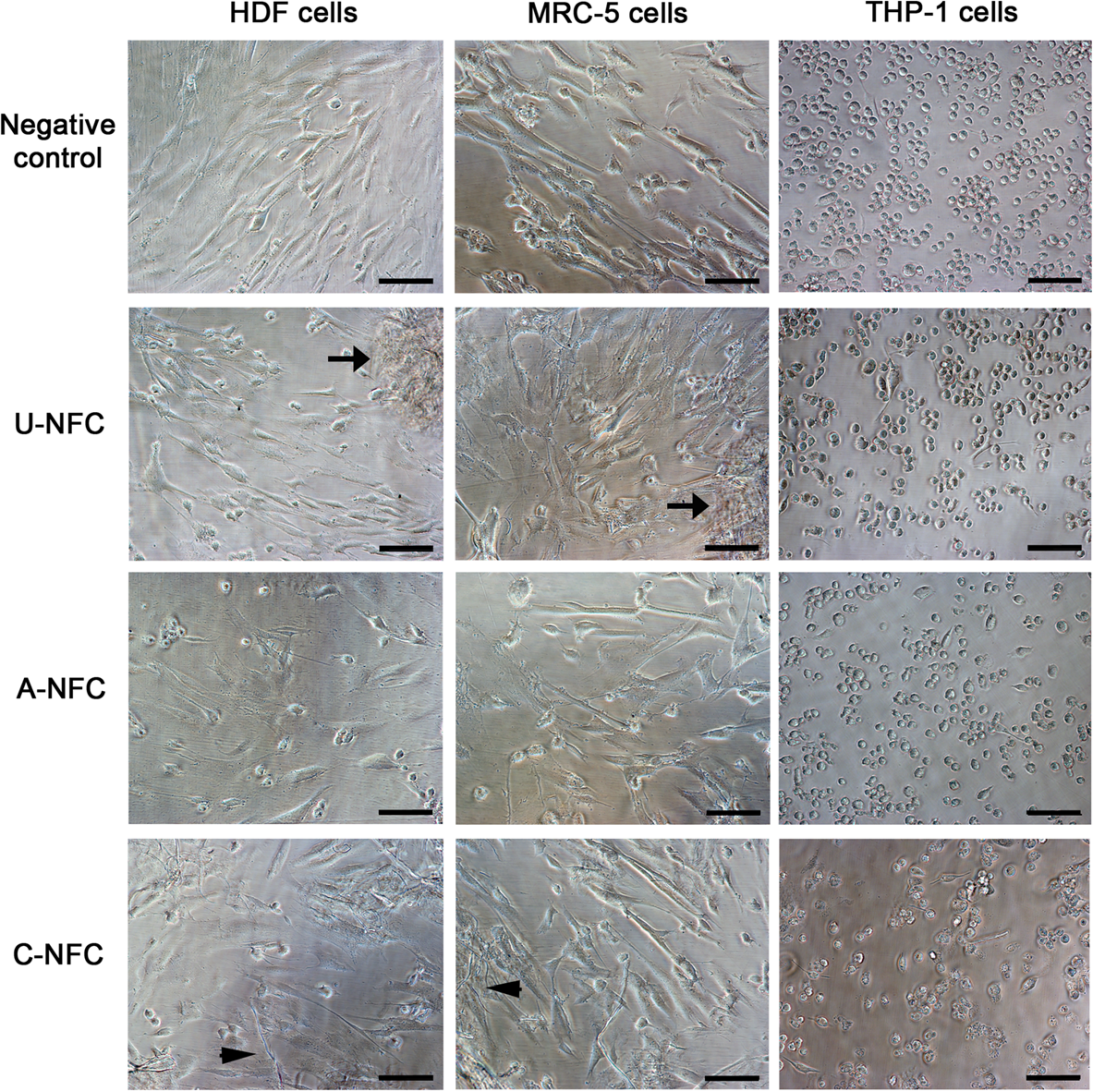
Cell Morphology after NFC exposure. Morphology of HDF, MRC-5 and THP-1 macrophages after direct contact with NFC. Top images show HDF, MRC-5 and THP-1 cells untreated (negative control). For all other conditions, HDF, MRC-5 and THP-1 cells were treated with the highest concentration of U-NFC, A-NFC and C-NFC (500 μg/mL) for 24 h. *Black* arrows indicate agglomerates of fibres. Images of cells treated with the positive control (DMSO 5%) are given in Additional file 1: Figure S1. Scale bars represent 100 μm

Accordingly, we did not find altered morphology for any of the three cell types treated with NFC gels after 24 h exposure when compared to the negative control (Fig. 5). In the positive control, 5% DMSO, cells presented unhealthy and round morphology, and loss of attachment, as expected (Additional file 1: Figure S1).

### U-NFC induces the release of pro-inflammatory cytokines

**Fig. 6:**
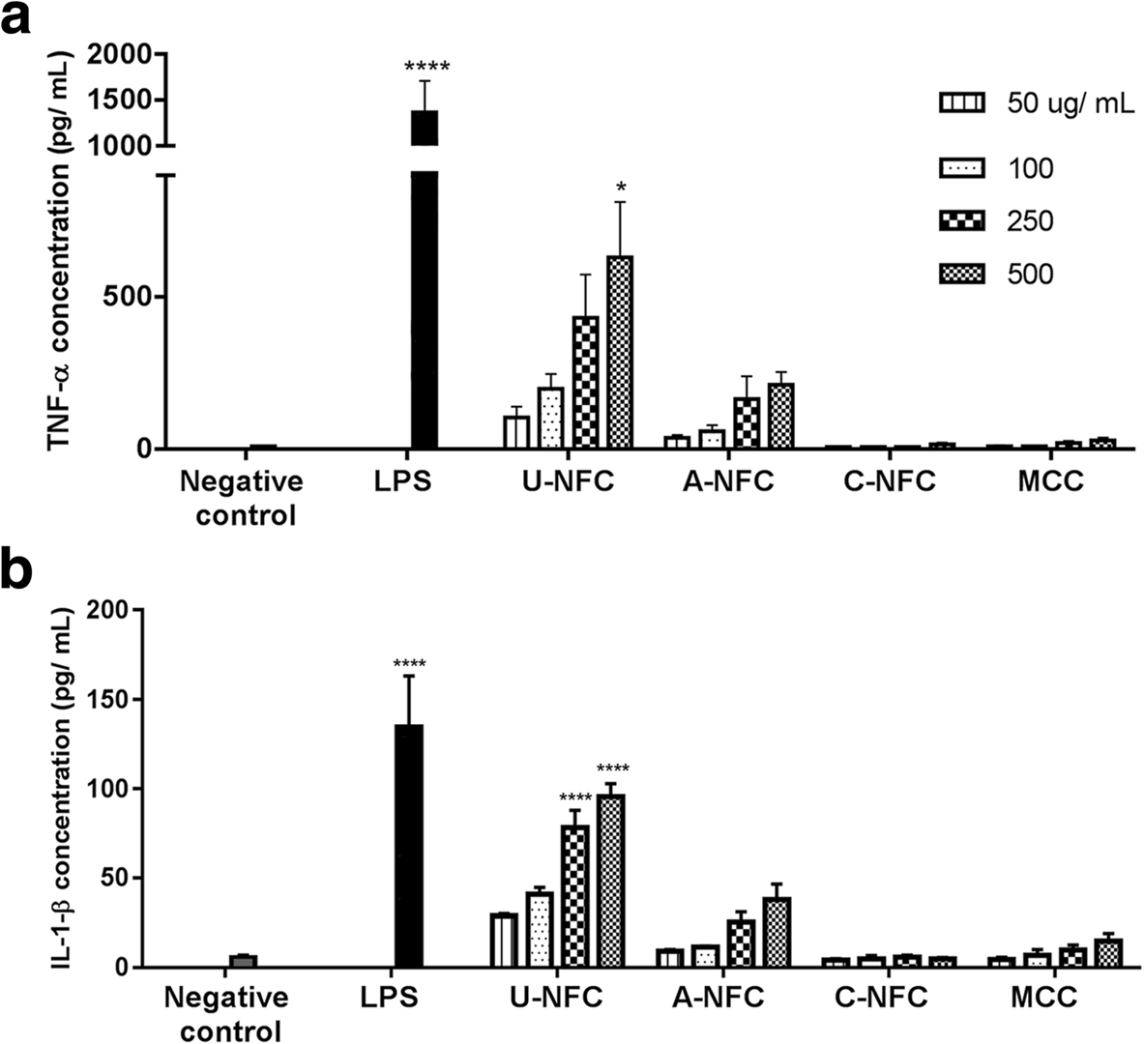
Cytokine production after NFC exposure. **a** TNF-α and **b** IL1-β concentration in the supernatants of THP-1 macrophages exposed to increasing doses of NFC (50–500 μg/mL) for 24 h. For the U-NFC sample cytokine secretion was assessed in the presence of PMB in order to supress the contribution of endotoxin contamination to the secreted cytokine levels. MCC is a commercial grade nanocellulose used as a reference material. Negative control represents untreated cells. Cells treated with LPS (1 ng/mL), an inducer of cytokine production, represent the positive control of the assay. The data are presented as mean ± SEM of three independent experiments. Significant results as compared to the negative control are marked with asterisks (*p < 0.05 and **** *p* < 0.0001)

Whether the NFC samples trigger an inflammatory response in THP-1 macrophages was evaluated by measuring the levels of two pro-inflammatory cytokines, TNF-α and IL1-β in cell culture supernatants following 24 h exposure. First, the possible input of NFC endotoxin contamination to cytokine production was investigated by measuring the cytokine levels in the presence of PMB. Results showed that the levels of cytokines secreted by cells treated with U-NFC were significantly higher in the absence of PMB than when the experiments were performed in the presence of the LPS inhibitor (Additional file 2: Figure S2A and S2B). Thus, indicating a contribution of endotoxin contamination to the inflammatory response trigger by U-NFC. Therefore, the inflammatory potential of such sample was further investigated by conducting the experiments in the presence of PMB in a concentration that was shown to inhibit cytokine secretion in LPS-stimulated THP-1 macrophages (Additional file 2: Figure S2C). In this way we assure that the secreted levels of TNF-α and IL-1β are solely a consequence of the material-cell interactions and not due to endotoxin contamination. A significant release of TNF-α by cells treated with high dose of U-NFC (500 μg/mL) compared with the negative control was observed after 24 h exposure. THP-1 macrophages treated with A-NFC or C-NFC did, however, not secrete significant levels of TNF-α compared to the negative control (Fig. 6a).

U-NFC triggered a significant release of IL1-β by THP-1 macrophages at the two high doses, 250 and 500 μg/mL (Fig. 6**b**). Both modified NFCs, A- and C-NFC, did not induce IL1-β secretion. MCC did not induce production of any of the pro-inflammatory cytokines under study.

### No intracellular ROS increase upon treatment with NFCs

**Fig. 7:**
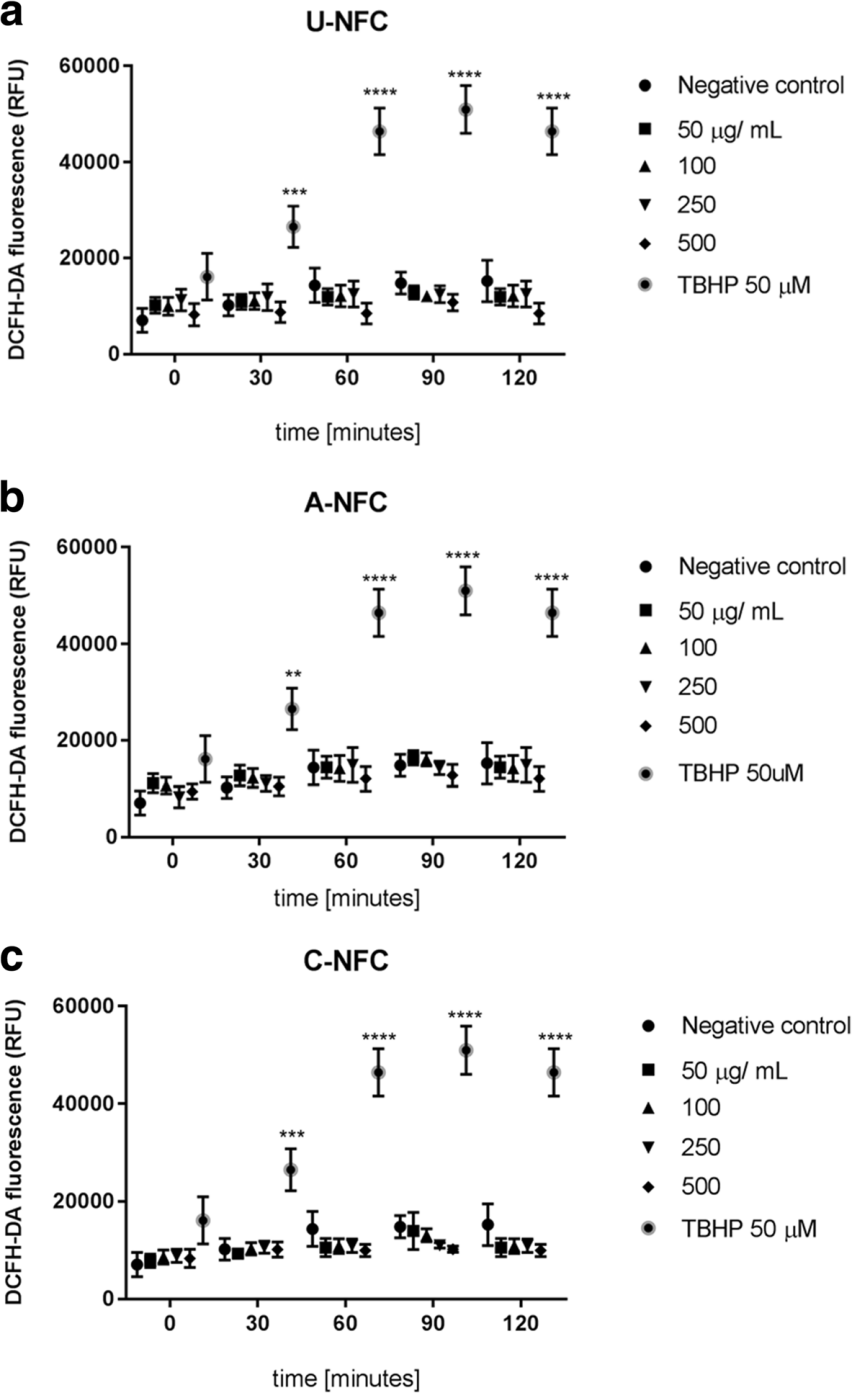
Cellular reactive oxygen species (ROS) production after NFC exposure. Kinetic study of ROS production of THP-1 macrophages treated with increasing doses (50–500 μg/mL) of (**a**) U-NFC, (**b**) A- NFC and (**c**) C-NFC. ROS assessed with the ROS-specific fluorescent probe DCFDA-DA every 30 min during 120 min. Negative control represents untreated cells. Tert-butyl hydroperoxide (TBHP), an inducer of oxidative stress, represents the positive control. Data are expressed as relative fluorescence units (RFU) and presented as the mean ± SEM of three independent experiments

For assessing the oxidative potential of NFCs, a kinetic study of intracellular ROS production was performed using the fluorescent marker DCFH-DA. No significant ROS increase was observed during the first 120 min for THP-1 macrophages treated with NFCs compared with the negative control (Fig. 7). The positive control, TBHP, induced a significant increase compared with the negative control. The reference material MCC displayed a similar cellular ROS profile as the investigated NFCs (see Additional file 3: Figure S3).

### THP-1 macrophages do not uptake the NFC materials

The TEM analysis of the cells after exposure to the different NFC materials confirmed that there were no alterations in cell morphology and showed that none of the NFC samples were uptaken by the THP-1 macrophages. No signs of phagocytosis attempts were found. Representative TEM images of THP-1 macrophages after 24 h exposure to the different NFC materials at a concentration of 500 μg/ml, together with images of the non-treated cells can be found in the supplementary information (Additional file 4: Figure S4).

## Discussion

In this study, the toxicity impacts of unmodified and surface modified NFC gels (i.e. carboxymethylated and hydroxypropyltrimethylammonium-substituted NFC) on dermal, lung and macrophage cells were compared for the first time. The selected human cell types represent cells most likely to be impacted by the potential exposure to NFC in an occupational setting, including immune surveillance and epithelial cells covering the respiratory tract and the skin.

Surface charges are introduced during the preparation of nanocellulose where adding repulsive charges facilitates the defibrillation process. As expected, the presence of surface charged groups in A-NFC and C-NFC and the subsequent electrostatic repulsion between fibres resulted in individual nanocellulose fibrils, while fibres tended to aggregate in the low carboxyl group content U-NFC. The zeta potential further confirmed the different surface charges of the studied samples, however in the presence of cell culture medium all samples showed slightly negative charge (zeta potential values between − 4 and − 12 mV). As described for other nanomaterials, the nanofibres may be rapidly covered by the biomolecules present in the cell culture medium, forming a biomolecular corona that partially or fully covers the nanofibers and mask the surface charge and, consequently, changes the zeta potential [26, 27].

The effect of protein adsorption was also reflected in the agglomeration pattern of A-NFC and C-NFC, which drastically changed from dispersed fibres in phosphate buffer to compact agglomerates in cell culture medium. As also observed by Tomic et al. [28], the agglomeration of U-NFC did not significantly change when comparing cell culture medium and phosphate buffer suspensions, showing the presence of small agglomerates in both conditions. The type, amount and conformation of the adsorbed proteins will be influenced by the nanofibre surface chemistry among other nanomaterials properties. In turn, the different protein adsorption patterns may promote distinct agglomeration states of the nanomaterial [29], as reflected here.

The assessment of potential toxic effects showed that the NFC materials under study did not have any impact on the metabolic activity or on the membrane integrity of the treated cells following 24 h exposure. Overall, the NFCs under study did not impair the cell viability of dermal, lung or macrophage cells. This is in accordance with previous in vitro studies showing that NFC gels are non- cytotoxic for a wide range of cells, including dendritic cells, macrophages, fibroblasts, keratinocytes, human cervic carcinoma and hepatoma cell lines [13–15, 28]. Moreover, NFC gels, aerogels and membranes were proven to be biocompatible when evaluated for diverse tissue engineering and biomedical applications [12, 18, 30–33].

Furthermore, no significant ROS production by THP-1 macrophages was found under the conditions tested in the present work. However, it was shown that U-NFC promoted an inflammatory response in terms of secretion of TNF-α and IL-1β, a response that was suppressed when surface charges were introduced on the nanofibrils.

It is well-known that the inhalation of toxic airborne particulates, particularly carbon nanotubes and other fibre-like nanomaterials can cause pulmonary inflammation [34]. Macrophages play a critical role in the recognition, and clearance of pathogens and foreigner particulates. The acute phase responses to inhaled particulates are described by a pulmonary inflammation set by the release of a number of inflammatory mediators, such as TNF-α and IL1-β. These two cytokines, TNF-α and IL1-β, acting synergistically, are involved in the pathogenesis of various acute and chronic respiratory diseases [35].

The secretion of pro-inflammatory cytokines by macrophages under the influence of NFC has been previously investigated in vitro and in vivo. While some authors showed that NFC gels were non pro-inflammatory in vitro [13, 15, 24, 31], Catalán and co-workers found a pro-inflammatory response to NFC in vivo [36]. However, the authors declared that the possible role of microbial contamination on the inflammatory effect could not be ruled out. Interestingly, when we have previously studied the pro-inflammatory response of THP-1 monocytes cultured on NFC films we found a pro-inflammatory effect with U-NFC and A-NFC, while C-NFC behaved as an inert material [24]. Thus, a pro-inflammatory effect was found when carboxymethyl groups (A-NFC) were introduced on the nanofibrils, an effect that was absent in the present work. Such difference in the inflammatory response of A-NFC could be partially due to the variations in the structures of the nanocelluloses under study, i.e. gels versus membranes, and in how the cells were exposed to the materials.

Hypothetical cellular mechanisms of inflammation caused by fibres include i) frustrated phagocytosis and the subsequent production of ROS and oxidative stress or ii) a direct effect of the fibres on the membrane receptors [37]. ROS production was not found when macrophages were incubated with the NFC gels studied here and TEM analysis of the exposed cells revealed no sings of phagocytosis attempts, with untreated cells and NFC-exposed cells showing comparable morphologies. Besides, it was not possible to detect the presence of any of the NFC materials inside the cells. Therefore, we hypothesize that the observed secretion of pro-inflammatory cytokines is likely a consequence of fibre-receptor interactions, where surface chemistry plays a key role. Surface chemistry influences the type, conformation and amount of adsorbed proteins which in turn mediate the cell-nanomaterial interactions [29]. It can be speculated that the protein corona of U-NFC might promote the interaction with THP-1 membrane receptors resulting in the secretion of pro-inflammatory cytokines. However, the presence of carboxymethyl and hydroxypropyltrimethylammonium groups on A-NFC and C-NFC, respectively, most probably result in protein coronas that do not promote the signalling for an inflammatory effect. Besides, surface chemistry and the subsequent protein adsorption might also indirectly affect the cellular response to the nanomaterial by impacting on its agglomeration pattern [29]. The difference in surface chemistry, particularly in surface charges, was indeed reflected in the agglomeration patterns of the NFC materials and therefore an effect of nanofibre dispersion on the observed inflammatory response cannot be dismissed. Authors have described the influence of fibre agglomerates in in vivo cellular responses of nanofibres such as carbon nanotubes and carbon fibres [38, 39]. Mutlu et al. showed that the aggregation of single-walled carbon nanotubes accounted for its pulmonary toxicity, an effect that could not be seen when the carbon nanotubes were nanoscale dispersed [39]. Nevertheless, in the present study, U-NFC with smaller agglomerates compared with A-NFC and C-NFC was the material that promoted a pro-inflammatory response and macrophages did not react differently to the diverse agglomerate sizes in terms of frustrated phagocytosis or cellular uptake.

The cellular uptake of NFC has recently been investigated by other authors [28, 36]. Catalán et al. administrated TEMPO-oxidized NFC (negatively charged NFC) to mice by pharyngeal aspiration and reported dose-related accumulation of the material in the cytoplasm of macrophages [36]. Conversely to our work, in vitro studies with dendritic cells showed partial internalization of NFC [28]. The authors stated that the interaction of NFC and the dendritic cells depended on the thickness and length of the material and highlight the need of further studies to investigate the mechanism that predominate in the NFC-dendritic cells interactions [28].

In summary, the findings presented here suggest that the inflammatory response to NFC gels might be driven by the surface chemistry. Furthermore, the fact that U-NFC induces an inflammatory response under no signs of toxicity may pose a concern and indicates that, as also observed by others, cytotoxicity and activity (e.g. inflammatory response) do not necessary correlate [40]. Understanding the effect of NFC features, in this case, the surface chemistry on their biological reactivity will contribute towards safer industrial applications of such nanomaterial. More studies are needed, especially for prolonged exposure times and in vivo tests, to further investigate the effect of NFC on the immune response and enhance our present knowledge about the safety of nanocelluloses.

## Conclusion

No indication of cytotoxicity or significant ROS production were found when cells were exposed to the unmodified and surface modified NFC gels. Besides, no cellular uptake was observed. A pro-inflammatory response with U-NFC in terms of cytokine secretion was found and this effect was suppressed when surface charged groups were present on the nanofibrils. This finding suggests that the inflammatory response to NFC gels might be driven by surface chemistry opening up the possibility for the design of safe nanocellulose materials.

## Additional files

Additional file 1: Figure S1. Light microscopy images of untreated (top panels) and DMSO-treatedd (bottom panels) cells. Top images show HDF, MRC-5 and THP-1 macrophage cells untreated (negative control) and bottom images show cells treated with DMSO 5% in cell culture media (positive control). Scale bars represent 100 μm. (TIF 4130 kb)
Additional file 2: Figure S2. Cytokines production after NFC exposure in the presence and absence of PMB. (A) TNF-α and (B) IL-1 β concentration in the culture supernatants of THP-1 macrophages exposed for 24 h with increasing doses of NFCs (50–500 μg/mL) with and without PMB-treatment. (C) Effect of PMB (25 μg/mL) on TNF-α and IL1-β production by THP-1 macrophages stimulated with LPS (1 ng/mL). Note that when PMB was added to the LPS treated cells, the cytokine secretion was reduced to a level comparable to that found for the negative control. MCC is a food grade nanocellulose used a as reference material. Negative control represents untreated cells. Data are presented as mean ± SEM of three independent experiments. Statistically significant differeces in cytokine secretion between PMB treated and untreated cells are marked with asterisks (** *p* < 0.01 and **** *p* < 0.0001). (TIF 389 kb)
Additional file 3: Figure S3. Cellular ROS production after the addition of MCC to THP-1 macrophages. Kinetic study of ROS production of cells treated with increasing doses (50–500 μg/mL) of MCC, a food grade nanocellulose. ROS assessed with the ROS-specific fluorescent probe DCFDA-DA every 30 min during 120 min. Negative control represents untreated cells. Tert-butyl hydroperoxide (TBHP), an inducer of oxidative stress, represents the positive control. Data are expressed as relative fluorescence units (RFU) and presented as mean ± SEM of three independent experiments. Significant results as compared to the negative control are marked with asterisks (*** *p* < 0.001 and **** *p* < 0.0001). (TIF 65 kb)
Additional file 4: Figure S4. Transmission electron microscopy analysis of THP-1 macrophages. Representative images of (a and b) untreated cells, (c and d) cells exposed to U-NFC, (e and d) cells exposed to A-NFC, (g and h) cells exposed to C-NFC. Cells were treated with 500 μg/ml of NFC for 24 h. The arrow indicate the presence of NFC agglomerates in the vicinity of the cells. (TIF 2251 kb)

## Abbreviations

AB: Alamar blue
A-NFC: Anionic nanofibrillated cellulose
ANOVA: Analysis of variance
C-NFC: Cationic nanofibrillated cellulose
DCF: Dichlorodihydrofluorescein
DCFH-DA: Dichlorodihydrofluorescein diacetate
DMEM/F12: Dulbecco’s Modified Eagle Medium: Nutrient Mixture F-12
DMSO: Dimethyl sulfoxide
ECACC: European Collection of Authenticated Cell Cultures
IL1-β: Interleukin 1 beta
LDH: Lactate Dehydrogenase
LPS: Lipopolysaccharide
NFC: Nanofibrillated cellulose
PBS: Phosphate buffer
PMA: Phorbol myristate acetate
PMB: Polymyxin B
ROS: Reactive oxygen species
RPMI 1640: Roswell Park Memorial Institute (RPMI) 1640 Medium
SEM: Standard error of the mean
TBHP: Tert-butyl hydroperoxide
TEM: Transmission electron microscopy
TNF-α: Tumor necrosis factor α
U-NFC: Unmodified cellulose nanofibrillated
