# Supplementary figures and images for: In vitro biological responses to nanofibrillated cellulose by human dermal, lung and immune cells: surface chemistry aspect
Source: Part Fibre Toxicol. 2017 Jan 10;14:1. doi: 10.1186/s12989-016-0182-0 (PMC5223298; doi:10.1186/s12989-016-0182-0)

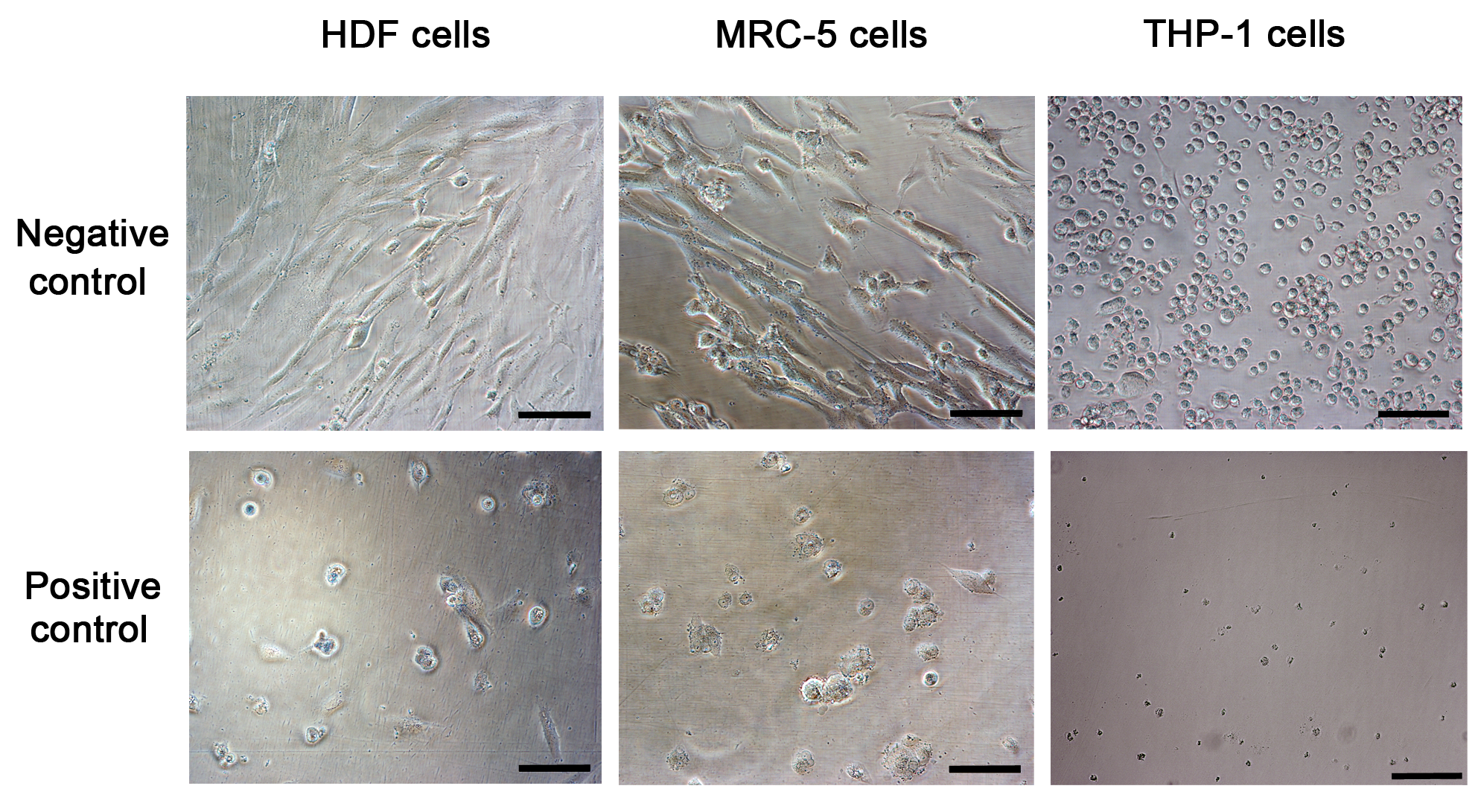

Supplement: Additional file 1: Figure S1. — Light microscopy images of untreated (top panels) and DMSO-treatedd (bottom panels) cells. Top images show HDF, MRC-5 and THP-1 macrophage cells untreated (negative control) and bottom images show cells treated with DMSO 5% in cell culture media (positive control). Scale bars represent 100 μm. (TIF 4130 kb) [file 12989_2016_182_MOESM1_ESM.tif]

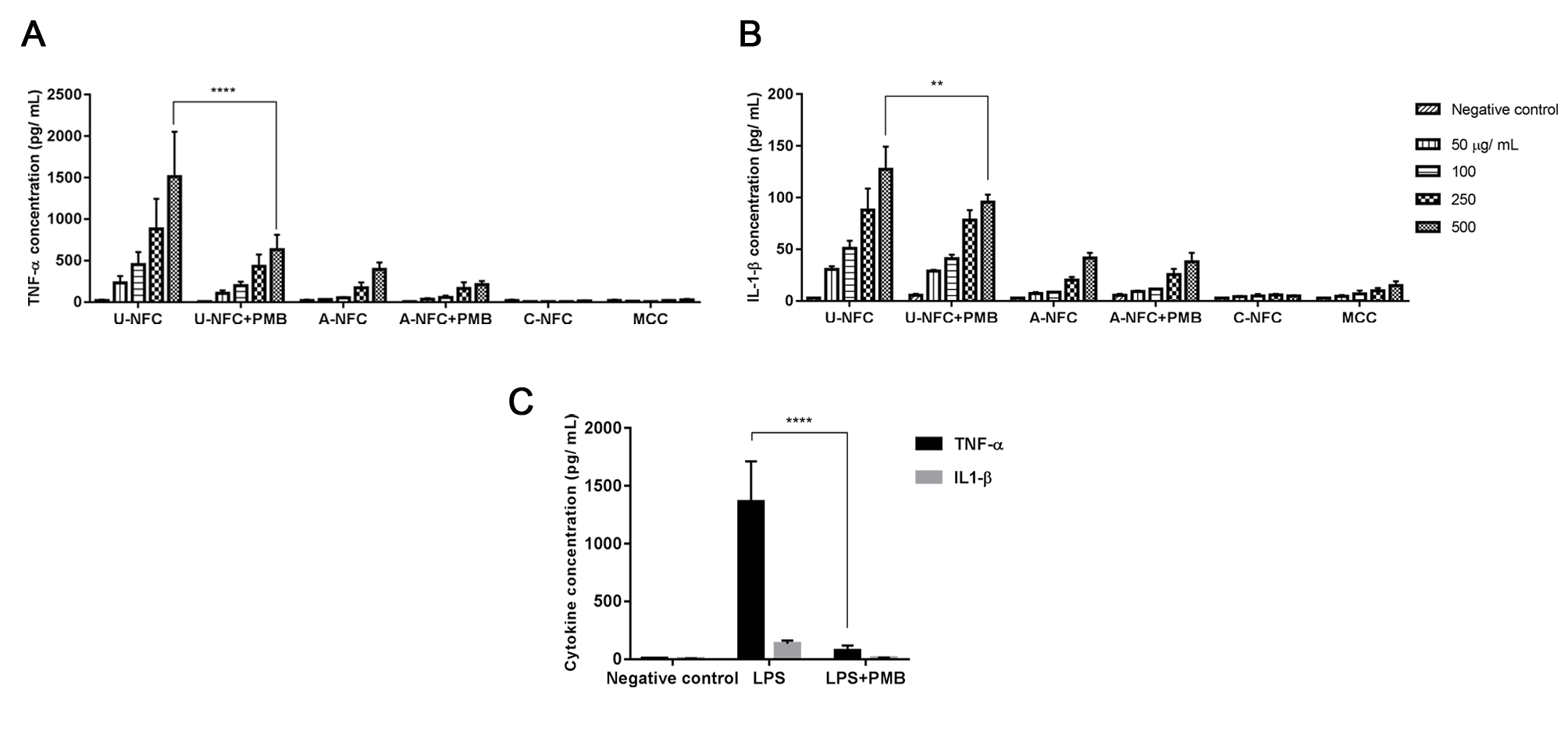

Supplement: Additional file 2: Figure S2. — Cytokines production after NFC exposure in the presence and absence of PMB. (A) TNF-α and (B) IL-1 β concentration in the culture supernatants of THP-1 macrophages exposed for 24 h with increasing doses of NFCs (50–500 μg/mL) with and without PMB-treatment. (C) Effect of PMB (25 μg/mL) on TNF-α and IL1-β production by THP-1 macrophages stimulated with LPS (1 ng/mL). Note that when PMB was added to the LPS treated cells, the cytokine secretion was reduced to a level comparable to that found for the negative control. MCC is a food grade nanocellulose used a as reference material. Negative control represents untreated cells. Data are presented as mean ± SEM of three independent experiments. Statistically significant differeces in cytokine secretion between PMB treated and untreated cells are marked with asterisks (** p < 0.01 and **** p < 0.0001). (TIF 389 kb) [file 12989_2016_182_MOESM2_ESM.tif]

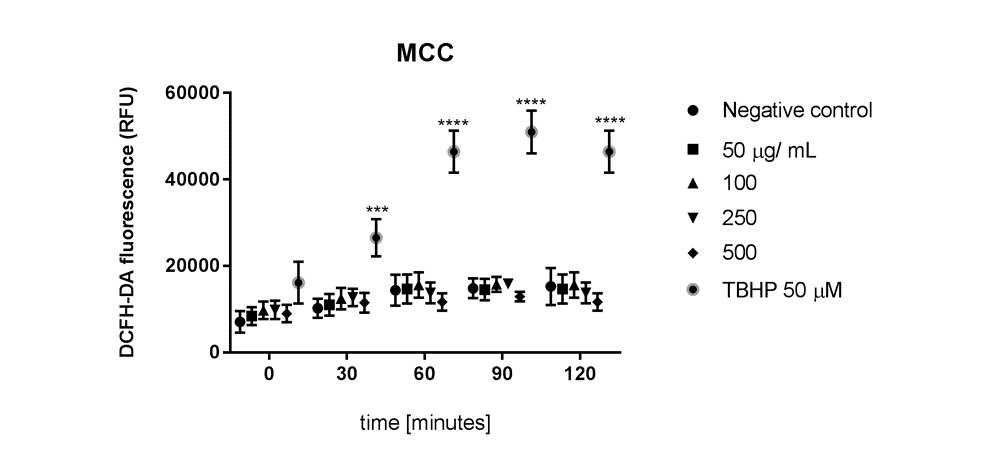

Supplement: Additional file 3: Figure S3. — Cellular ROS production after the addition of MCC to THP-1 macrophages. Kinetic study of ROS production of cells treated with increasing doses (50–500 μg/mL) of MCC, a food grade nanocellulose. ROS assessed with the ROS-specific fluorescent probe DCFDA-DA every 30 min during 120 min. Negative control represents untreated cells. Tert-butyl hydroperoxide (TBHP), an inducer of oxidative stress, represents the positive control. Data are expressed as relative fluorescence units (RFU) and presented as mean ± SEM of three independent experiments. Significant results as compared to the negative control are marked with asterisks (*** p < 0.001 and **** p < 0.0001). (TIF 65 kb) [file 12989_2016_182_MOESM3_ESM.tif]

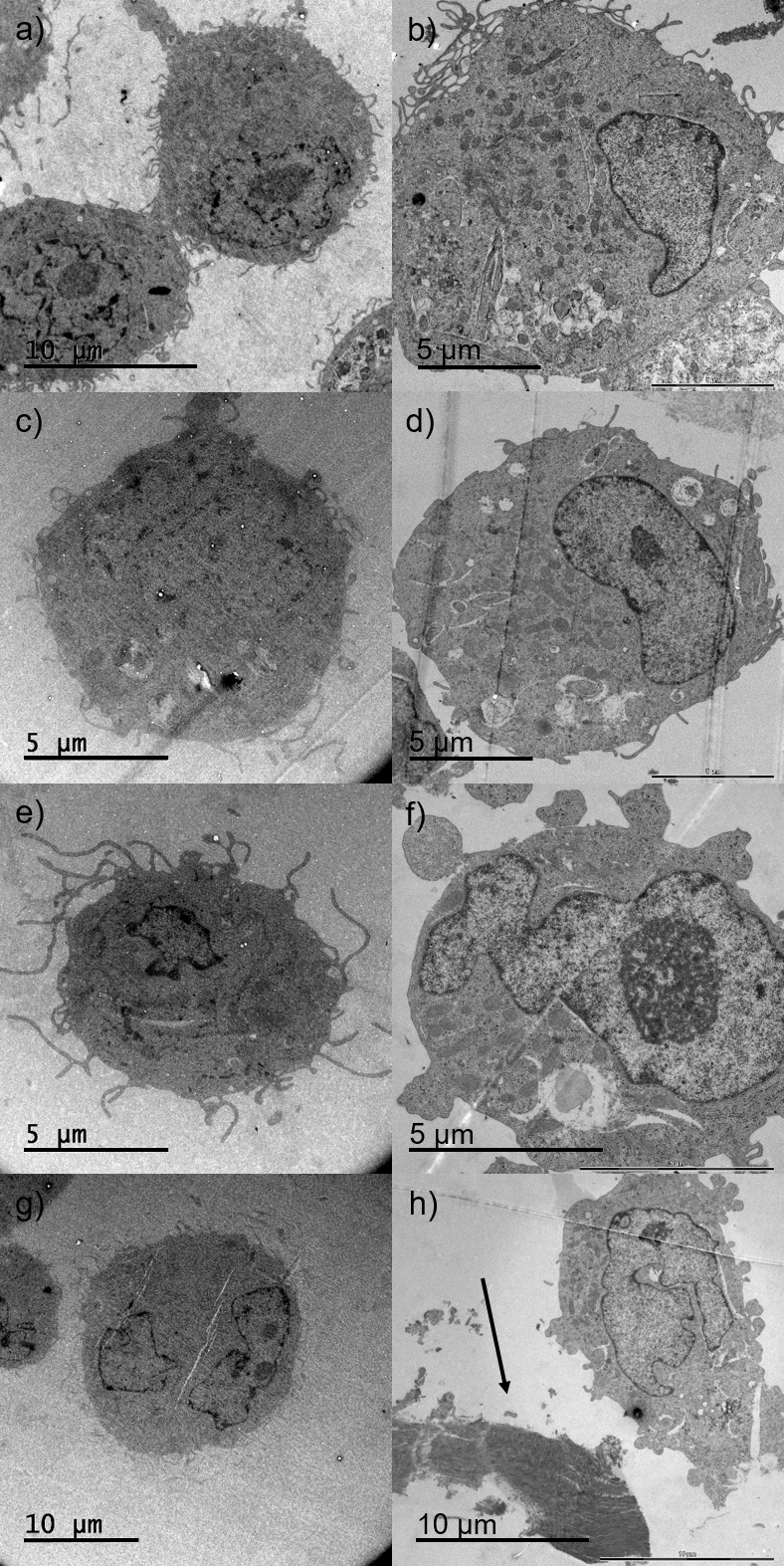

Supplement: Additional file 4: Figure S4. — Transmission electron microscopy analysis of THP-1 macrophages. Representative images of (a and b) untreated cells, (c and d) cells exposed to U-NFC, (e and d) cells exposed to A-NFC, (g and h) cells exposed to C-NFC. Cells were treated with 500 μg/ml of NFC for 24 h. The arrow indicate the presence of NFC agglomerates in the vicinity of the cells. (TIF 2251 kb) [file 12989_2016_182_MOESM4_ESM.tif]
